# Trends in sugar-sweetened beverage prices, sales, and elasticities: policy evidence from WHO regions, 2010–2024

**DOI:** 10.3389/fpubh.2026.1782007

**Published:** 2026-04-09

**Authors:** Hussain Ali, Rong Zheng, Yuxiao Hu, Saamiya Humayon

**Affiliations:** 1 University of International Business and Economics, Beijing, China; 2 Lahore University of Management Sciences, Lahore, Pakistan

**Keywords:** fiscal policy, price elasticity, price trend, sales trend, sugar-sweetened beverages, WHO regions

## Abstract

Sugar-sweetened beverages (SSBs) are a major source of added sugars and an important focus for fiscal policies aimed at preventing obesity, type 2 diabetes, and other non-communicable diseases (NCDs). We aimed to assess trends in sugar-sweetened beverage sales and inflation-adjusted prices and to estimate the price elasticity of demand across World Health Organization (WHO) regions from 2010 to 2024, providing evidence to support fiscal policies for health. Annual country-level data on sugar-sweetened beverage sales volumes and values for 93 geographic locations, representing 81.74% of the 2024 global population, were obtained from the Euromonitor International Passport database and grouped into six WHO regions. Sales values were converted to international dollars using purchasing power parity and adjusted for inflation, and regional average real prices were calculated. Trends in sales volumes, sales values, and prices were analysed using joinpoint regression. Price elasticities of demand were estimated using a baseline panel regression model of the log of sales volume per capita on the log of real price, controlling for macroeconomic indicators, with regional elasticities derived from the model’s marginal effects, and an instrumental variables specification was used for robustness. Between 2010 and 2024, the global sugar-sweetened beverage sales volume increased by 16.9%, with the largest rises in Southeast Asia (101.6%) and Africa (81.5%) and smaller increases in the Americas, the Western Pacific Region, the Eastern Mediterranean, and Europe. The global price elasticity of demand was −0.423 (*p* < 0.01). It was highest in Europe (−0.828, *p* < 0.01) and the Americas (−0.509, *p* < 0.05), while elasticities in other regions were not statistically significant. The substantial increase in SSB sales in emerging markets, such as Southeast Asia and Africa, along with a moderate increase in other regions, indicates the need for comprehensive SSB control policies and the implementation of WHO SSB taxation policies (2022). The divergent regional trajectories highlight the need for comprehensive fiscal and regulatory policies to curb the growth in SSB consumption and encourage shifts towards healthier options.

## Introduction

Sugar-sweetened beverages (SSBs), including, soft drinks, sweetened juices, energy drinks, and sweetened teas and coffees, are loaded with added sugars and calories, contributing to excessive caloric intake without providing important nutrients (1–3). Global consumption of sugar-sweetened beverages (SSBs) has increased substantially over the past two decades, contributing to rising rates of obesity, type 2 diabetes, and other non-communicable diseases (NCDs) worldwide (4–9). Despite recent policy actions in various geographic locations, global consumption of SSBs remains well above recommended levels, and sales continue to expand in several low- and middle-income regions (3, 10). The increase in sales of SSBs is attributed largely to increasing population, dietary transitions, rapid urbanisation, rising disposable incomes in low- and middle-income countries, and aggressive industry marketing, with efforts to ensure that these products remain highly affordable and accessible (11–13).

The World Health Organization (WHO) has urged countries to introduce or strengthen taxes on SSBs as a core fiscal measure to improve public health and reduce the prevalence of NCDs (14). The WHO recommends excise taxes that increase the retail prices of SSBs by at least 20% to reduce consumption (15). Evidence from policy evaluations and reviews indicates that SSB taxes are often reflected in consumer prices and are associated with reductions in both the sales of and intake of these beverages (16). Emerging evidence indicates that SSB taxes can increase the prices of taxed beverages, reduce sales and purchases, and generate revenues that may be reinvested in community and health-promoting programmes (17–19). A 20% increase in the price of all SSB categories would reduce the consumption of SSBs and increase the consumption of non-SSBs, as per a survey-based study, and this effect is strengthened when sugar content is clearly labelled on the product (20). Evidence synthesised from implemented SSB taxes worldwide indicates that such taxes are associated with higher prices for taxed beverages and meaningful reductions in sales (21). The global effort to reduce the consumption of SSBs aligns with the United Nations Sustainable Development Goal (SDG) 3.4, which aims to reduce premature mortality from NCDs by one-third by 2030 (22, 23).

This study examines trends in SSB sales volumes, inflation-adjusted sales values, and inflation-adjusted average prices across WHO regions for 2010–2024 and estimates regional price elasticities. Price elasticities of SSB intake represent the percentage change in intake resulting from a 1% change in price (24). WHO regional sales trends and price elasticities may be highly important for planning control policies and fiscal policies (25). They provide a better understanding of the implications of SSB taxes (as recommended by the WHO) and highlight regional differences. These findings directly support the strengthening of fiscal policies, such as SSB taxation, to reduce consumption in line with global NCD prevention goals.

As a conceptual framework for this study, price-raising policies such as excise taxes (consistent with the WHO guidance recommending tax designs that raise SSB retail prices by at least 20%) are intended to increase consumer-facing SSB prices, thereby reducing demand according to the price elasticity of consumption. A decrease in demand translates into reduced purchases and lower consumption of SSBs, thereby lowering excess sugar and energy intake at the population level. Over time, reduced sugar intake is expected to lower the risk of obesity, type 2 diabetes, and other diet-related NCDs, thereby supporting progress towards Sustainable Development Goal 3.4. Accordingly, estimating trends in prices and demand responsiveness provides policy-relevant evidence on the expected direction and magnitude of consumption changes under fiscal measures.

### Policy implications

- Global sales of sugar-sweetened beverages increased by 16.9% from 2010 to 2024, with the largest rises observed in Africa (81.5%) and Southeast Asia (101.6%). Sales volumes also increased in all other regions.
- Demand is price-responsive globally, with a price elasticity of −0.423 (*p* < 0.01), indicating that price increases can reduce SSB consumption, although regional disparities across WHO regions remain.
- Global SSB consumption continues to rise, indicating that current taxes, prices, and control policies are insufficient.
- These findings provide evidence to support WHO-recommended fiscal policies, including stronger SSB taxation, to curb intake and advance Sustainable Development Goal 3.4.
- Regional demand elasticities can help guide effective fiscal policies, such as SSB taxes tailored to local consumption patterns.

## Methods

### Data

We operationalised SSBs using Euromonitor Passport’s soft drinks market aggregate. Accordingly, sales data for SSBs from 2010 to 2024 were retrieved from the Euromonitor International Passport database, available under the category of “soft drinks.” Within this category, we included the following subcategories that constitute SSBs: Asian Speciality Drinks, Concentrates, Energy Drinks, Juice, Ready-to-Drink (RTD) Coffee, RTD Tea, Regular Carbonates, and Sports Drinks. We excluded bottled water from our analysis. Euromonitor aggregates several beverage groups under the “soft drinks” category and does not consistently provide a separate series that allows the exclusion of 100% fruit juice or low-sugar variants across all geographic locations and years. Therefore, our SSB measure reflects the broader soft drinks market as captured by Euromonitor (excluding bottled water), which may include some 100% juices and low-sugar variants. Accordingly, our measure should be interpreted as the soft drink market (excluding bottled water), serving as a consistent proxy for SSB-related beverage demand in cross-country trend and elasticity analyses (26). Euromonitor compiled these statistics using expert interviews and research from multiple sources, including official publications, industry reports, scientific journals, and online resources (27).

Initially, the total number of geographic locations included in the study was 98, of which 5 were removed due to missing information. The remaining 93 geographic locations were grouped into 6 WHO regions: Africa (AFRO; *n* = 10), the Americas (AMRO; *n* = 16), Europe(EURO; *n* = 37), Southeast Asia (SEARO; *n* = 6), the Eastern Mediterranean (EMRO; *n* = 12), and the Western Pacific Region (WPRO; *n* = 12), collectively representing 81.74% of the 2024 global population (28). Another category of “global” was created to combine all geographic locations.

### Measures

The data included annual SSB sales volumes (litres) and sales values (local currency) for the years 2010–2024, covering the following categories: Asian Speciality Drinks, Concentrates, Energy Drinks, Juice, Ready-to-Drink (RTD) Coffee, RTD Tea, Regular Carbonates, and Sports Drinks. For each country and year from 2010 to 2024, we summed the sales volumes and sales values across all SSB categories to calculate the total annual SSB sales volume and sales value. Sales values, representing industry revenue, were converted to international dollars ($Intl) using purchasing power parity for each country and year (29). They were then adjusted for inflation using the USA Consumer Price Index (2010 = 100). Finally, total SSB sales volumes and sales values were aggregated by WHO region. To capture pricing strategies and tax effects, the inflation-adjusted average price per litre ($Intl) was calculated by dividing each country’s inflation-adjusted sales values ($Intl) by the total SSB sales volume in litres. The average real price in international dollars for each WHO region and year was calculated by dividing the total regional sales value by the total regional sales volume for that year.

### Analysis

Percentage changes in all metrics from 2010 to 2024 were reported overall and by region. Statistical significance was tested using joinpoint regression (version 5.4.0.0) to estimate the average annual percentage change (AAPC, *p* < 0.05).

We used a baseline panel regression model to estimate the SSB price elasticity. STATA 18 software was used for regressions. We regressed the log of SSB sales volume for each country on the log of inflation-adjusted price, controlling for macroeconomic indicators, including gross domestic product (GDP) (30) per capita (constant $Intl) and inflation. Price elasticities for WHO regions were derived from the model’s marginal effects. For robustness, we performed an instrumental variables regression using the first lag of the independent variable as the instrumental variable.

## Results

### Trends in sales and prices

**Figure 1:**
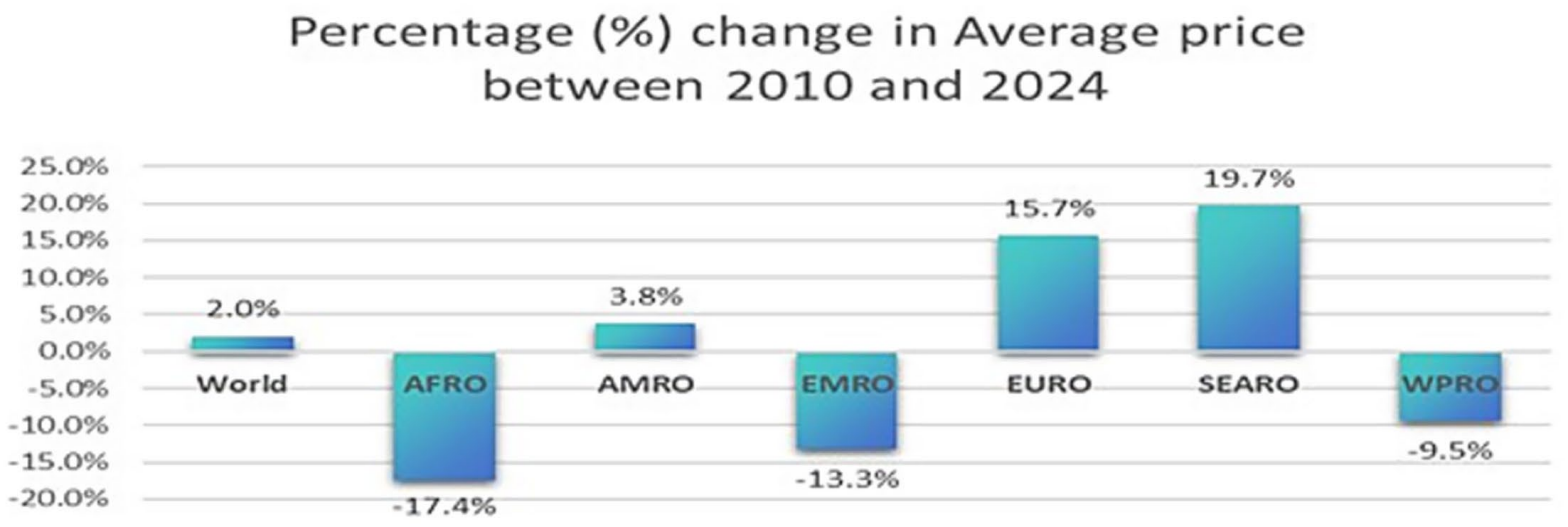
Percentage (%) change in the average price between 2010 and 2024.

**Figure 2:**
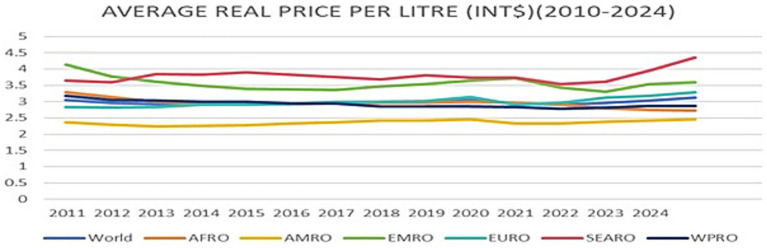
Average real price per litre (Int$; 2010–2024).

**Figure 3:**
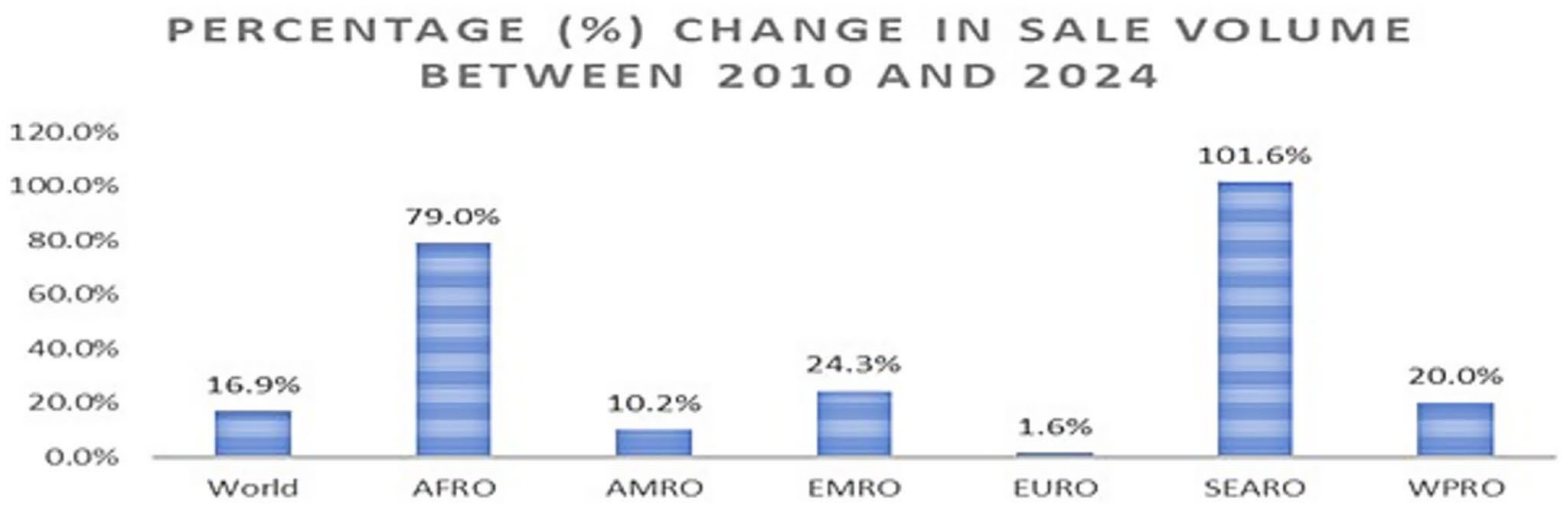
Percentage (%) change in the sales volume between 2010 and 2024.

**Figure 4:**
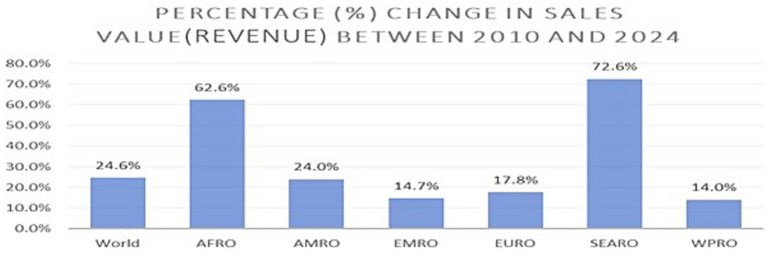
Percentage (%) change in the sales value (revenue) between 2010 and 2024.

Figures 1–4 illustrate trends in SSB sales and prices across WHO regions (2010–2024). SSB sales data for 93 geographic locations from 2010 to 2024 were obtained from the proprietary Euromonitor International Passport database. Geographic locations were divided into six WHO regions: Africa (*n* = 10), the Americas (*n* = 16), Europe (*n* = 37), Southeast Asia (*n* = 6), the Eastern Mediterranean (*n* = 12), and the Western Pacific Region (*n* = 12), collectively representing 81.74% of the global population in 2024 (28). Another category of “world” was created to combine all geographic locations.

As shown in Figure 1, between 2010 and 2024, the inflation-adjusted average SSB price per litre increased globally by only 2% (AAPC = 0.17). Regions with notable price increases included Southeast Asia (19.7%, AAPC = 0.28), the Americas (3.8%, AAPC = 0.43), and Europe (15.7%, AAPC = 0.83*). Regions that showed a significant decrease in the inflation-adjusted mean price from 2010 to 2024 included Africa (−17.4%, AAPC = -1.39*), the Eastern Mediterranean (−13.3%, AAPC = -0.52), and the Western Pacific Region (−9.5%, AAPC = -0.75*). “*” indicates that the AAPC is significantly different from zero at alpha = 0.05.

Figure 2 illustrates the average real price per litre in international dollars from 2010 to 2024 across WHO regions. In 2024, the inflation-adjusted average price per litre (Intl$) of SSBs in Africa (Int$2.72) and the Western Pacific Region (Int$2.88) remained below the global average of Int$3.11. Prices in the Region of the Americas (Int$2.45) were also below the global level. In contrast, Europe (Int$3.23), Southeast Asia (Int$4.36), and the Eastern Mediterranean (Int$3.59) recorded prices above the global average.

Figure 3 illustrates the percentage (%) change in SSB sales volume between 2010 and 2024 across WHO regions. The sales volume increased substantially in all regions during this period. Globally, it increased by 16.9% (AAPC = 1.11*), with the largest increases observed in Africa (79%, AAPC = 4.36*) and Southeast Asia (101.6%, AAPC = 4.68*). The sales volume also increased in all other regions, including the Americas (10.2%, AAPC = 0.78*), the Western Pacific Region (20%, AAPC = 1.36*), Europe (1.6%, AAPC = 0.12), and the Eastern Mediterranean (24.3%, AAPC = 1.61*).

Figure 4 shows the percentage (%) change in sales values (Revenue) between 2010 and 2024. Similar to trends in the sales volume and inflation-adjusted average price of SSBs, inflation-adjusted sales values (revenue) increased globally by 24.6% (AAPC = 1.65*), driven by significant increases in Southeast Asia (72.6%, AAPC = 3.84*), Africa (62.6%, AAPC = 3.42*), the Americas (24.0%, AAPC = 1.62*), the Eastern Mediterranean (14.7%, AAPC = 0.98*), Europe (17.8%, AAPC = 1.31*), and the Western Pacific Region (14%, AAPC = 1.04*). Changes in sales values across all regions were statistically significant.

### SSB price elasticities

Estimates from the baseline panel regression model largely aligned with those from the instrumented model (Tables 1, 2). The baseline model reflects the price responsiveness of SSB demand across WHO regions. The global price elasticity of demand was −0.423 (*p* < 0.01), indicating that a 1% price increase would lead to a 0.423% decline in SSB sales per capita. Price elasticities varied across regions, with the highest observed in Europe (−0.828, *p* < 0.01), followed by the Americas (−0.509, *p* < 0.05). In Africa (−0.090), the Eastern Mediterranean (−0.01), Southeast Asia (−0.031) and the Western Pacific Region (−0.202), the results were not statistically significant. Non-significant elasticities in some WHO regions may reflect low price variation, inconsistent SSB taxation, or high affordability over time, as these factors are commonly documented to reduce the statistical detectability of price effects (31, 32). It is worth noting that the baseline regression model for the African region was not statistically significant, while the instrumental variables model produced significant results. This could be due to the lagged period affecting the sample size, which, in turn, influenced statistical significance.

**Table 1:** Baseline regression.

Variables	(1) AFRO	(2) AMRO	(3) EMRO	(4) EURO	(5) SEARO	(6) WPRO	(7) Worldwide
	lnVolume	lnVolume	lnVolume	lnVolume	lnVolume	lnVolume	lnVolume
lnPrice	−0.090	−0.509**	−0.01	−0.828***	−0.031	−0.202	−0.423***
	(0.107)	(0.236)	(0.317)	(0.234)	(0.165)	(0.471)	(0.127)
_cons	1.758	−7.004	6.578	−11.986*	−3.938	−24.060***	−8.045***
	(2.575)	(4.868)	(4.800)	(6.821)	(3.368)	(7.422)	(2.856)
Controls	Yes	Yes	Yes	Yes	Yes	Yes	Yes
Time FE	Yes	Yes	Yes	Yes	Yes	Yes	Yes
Country FE	Yes	Yes	Yes	Yes	Yes	Yes	Yes
Observations	150	240	177	551	84	178	1380
R-squared	0.634	0.496	0.352	0.419	0.905	0.553	0.304

Standard errors are in parentheses.

***p < 0.01; **p < 0.05; *p < 0.1.

**Table 2:** Instrumental variables regression.

Variables	(1) AFRO	(2) AMRO	(3) EMRO	(4) EURO	(5) SEARO	(6) WPRO	(7) Worldwide
	lnVolume	lnVolume	lnVolume	lnVolume	lnVolume	lnVolume	lnVolume
lnPrice	−0.168**	−0.522***	−0.234	−1.078***	−0.038	−0.127	−0.547***
	(0.074)	(0.092)	(0.287)	(0.079)	(0.082)	(0.222)	(0.048)
_cons	2.151	−8.742***	5.196	−11.286***	−3.406	−21.729***	−7.313***
	(1.459)	(2.203)	(3.031)	(1.640)	(2.384)	(2.852)	(0.838)
Controls	Yes	Yes	Yes	Yes	Yes	Yes	Yes
Time FE	Yes	Yes	Yes	Yes	Yes	Yes	Yes
Country FE	Yes	Yes	Yes	Yes	Yes	Yes	Yes
Observations	140	224	165	515	78	166	1288
R-squared	0.630	0.507	0.337	0.387	0.878	0.496	0.259

Using the first lag of the independent variable as the instrumental variable.

## Discussion

Global SSB sales volumes, sales values, and prices have all increased over the past decade, albeit to varying degrees. Regional trends show significant differences between the regions. The global inflation-adjusted price increased slightly by 2% in 2024 compared to 2010. This minimal average price rise highlights the potential to further implement and strengthen SSB taxes, as recommended by the WHO (14). The global sales volume, rather than reducing in line with the United Nations Sustainable Development Goal (SDG) 3.4 (22), has increased by 16.9%. This trend is consistent with global dietary evidence showing that, despite worldwide efforts, adult consumption of SSBs rose by approximately 16% between 1990 and 2018 (33). Regional trajectories, however, continued to differ substantially. The Southeast Asia and European regions saw increases in inflation-adjusted average prices alongside rising inflation-adjusted sales values, whereas inflation-adjusted average prices declined in Africa, the Eastern Mediterranean, and the Western Pacific Region.

SSB control efforts and fiscal measures are still emerging and evolving (34). This is also reflected in our results, which show heterogeneity in pricing environments across WHO regions and highlight the uneven implementation of SSB taxation policies worldwide. The WHO recommends raising SSB prices by at least 20% to meaningfully influence consumption patterns and reduce the risk of NCDs (14, 15, 35).

Sales volumes increased across all WHO regions, with the strongest growth observed in Africa and Southeast Asia. This is consistent with international evidence showing faster soft drinks market growth and nutrition-transition dynamics in low- and middle-income regions compared to more mature markets (3, 36). Europe, by contrast, experienced an increase of only 1.6% in SSB sales volumes, the smallest among all regions. These regional differences are supported by other international SSB market studies showing faster consumption growth in low- and middle-income regions and slower demand in more mature markets (3). The increasing trend in sales volumes in all WHO regions highlights the need for stronger implementation and enforcement of SSB taxation. A comprehensive SSB control plan, including labelling measures, could further strengthen the effect of fiscal measures to curb SSB demand (20).

Similar to SSB sales volume trends, sales values increased across all regions. This highlights the need to increase the overall average real price according to WHO SSB taxation guidelines (6, 14, 20, 35) to counter the effects of expanding markets. However, our results reflect a concerning trend in the average real price of SSBs, where they remain highly affordable in most geographic locations. Inflation-adjusted average real prices (from 2010 to 2024) declined in Africa, the Eastern Mediterranean, and the Western Pacific Region. The Americas showed only a modest increase in inflation-adjusted SSB prices (3.8% in 2024 vs. 2010), suggesting there is still room to strengthen price-raising measures such as excise taxes. This interpretation aligns with evidence from Latin America and the Caribbean showing that SSB excise taxes are often a relatively small share of retail prices, highlighting the potential to increase tax levels and improve tax design to achieve larger price increases (37). The Southeast Asia region, despite a 19.7% increase in the average real price per litre, experienced the largest growth in the SSB sales volume. This pattern is plausible in settings undergoing rapid income growth and market expansion, where rising affordability can offset the dampening effects of higher prices (11). The WHO (2022) notes that SSB taxes are most effective when implemented as part of a broader, comprehensive strategy alongside other diet and NCD interventions (14). Hence, comprehensive SSB control policies may be needed, as fiscal measures alone may not be sufficient to curb consumption.

For the regional price elasticity of demand for SSBs, our estimated global price elasticity was −0.423 (*p* < 0.01), indicating that a 1% price increase would lead to a 0.423% decline in SSB sales per capita. This global estimate (−0.423) is directionally consistent, but smaller in magnitude, than pooled estimates from meta-analyses of implemented SSB taxes, which report tax elasticities around −1.00 (38). These differences are plausible because our outcome used a harmonised Euromonitor “soft drinks (excluding bottled water)” proxy for cross-country comparability, which may include some non-SSB components and attenuate elasticity towards zero. In addition, it is also because macro panel estimates capture broader market-level variation rather than short-run consumer responses to discrete tax-induced price shocks. By comparison, using cross-country macro panel data on market sales and prices, Goryakin et al. reported a global log–log elasticity of approximately −0.21 and around −0.32 in higher-income settings (39). Our price elasticity estimations varied across regions, with the highest observed in Europe (−0.828, *p* < 0.01), followed by the Americas (−0.509, *p* < 0.05). In Africa (−0.090), the Eastern Mediterranean (−0.01), Southeast Asia (−0.031), and the Western Pacific Region (−0.202), the results were not statistically significant. A WHO-recommended 20% price increase would be expected to reduce global consumption by approximately 8.5%. The implied reductions vary by region, ranging from approximately 16.6% in Europe and 10.2% in the Americas to smaller expected changes in other regions (e.g., 4.0% in the Western Pacific Region and 1.8% in Africa). These regional elasticity results are consistent with cross-country analyses reporting greater price responsiveness in higher-income groups, which supports our relatively larger elasticities in Europe and the Americas compared to Africa and Southeast Asia (24). Low-price variation, inconsistent SSB taxation, or high affordability over time may reduce the statistical detectability of price effects, causing significant elasticities in some WHO regions (31).

The sharp growth in SSB sales volumes in Africa, combined with declining real prices, highlights a widening gap between current policy efforts and WHO recommendations. The WHO’s 2022 fiscal policy guidance explicitly urges countries to implement excise taxes that meaningfully increase SSB prices as part of global NCD prevention strategies aligned with SDG 3.4 (14). Our results indicate that several regions are moving in the opposite direction, with declining real prices and rising consumption. Strengthening SSB taxation, monitoring affordability, and implementing other control policies may be critical for reducing SSB consumption and addressing the associated NCD burden (40).

Some limitations of this study must be acknowledged. First, Euromonitor data, although widely used, may be subject to methodological uncertainties, and independent verification at the country level remains limited. Informal market consumption in low- and middle-income countries may not have been fully captured, potentially affecting regional estimates. Nonetheless, Euromonitor remains the most comprehensive, harmonised global source for soft drink sales data, particularly in low- and middle-income countries and geographic locations. Second, category-specific SSB data were not analysed, which may have resulted in a general estimate of SSB pricing per litre rather than prices specific to each category. Future research could examine trends and elasticities for each SSB subcategory. Although our dataset covers 93 geographic locations representing more than 81.74% of the global population, trends may differ in countries or territories not included in the analysis. Third, our measure is based on Euromonitor’s soft drinks aggregate (excluding bottled water) and may include some beverages outside the strict WHO SSB definition (e.g., 100% juice or low-sugar variants), which could attenuate elasticity estimates towards zero. Although this approach is commonly used in cross-country trend and elasticity research using Euromonitor data, it remains a limitation. Finally, although regional data may provide a broad understanding of the situation, they cannot reveal country-specific factors driving these results. Therefore, country-level studies may be required to identify the causes of the regional heterogeneity and disparities we observed.

## Conclusion

This study provides one of the most comprehensive assessments to date of global trends in sugar-sweetened beverage prices, sales volumes, sales values, and price elasticities across 93 geographic locations within WHO regions from 2010 to 2024. Together, these findings highlight the need for stronger, sustained SSB taxation that raises real prices, reduces affordability, and supports healthier consumption patterns, in line with the WHO fiscal guidance and global NCD prevention goals.

## Data Availability

The data analyzed in this study were obtained from Euromonitor International Passport through institutional access and are not publicly available due to third-party restrictions. Access to the underlying data can be obtained directly from Euromonitor, subject to their terms and conditions. The analysis code and non-proprietary derived outputs supporting the conclusions of this article will be made available by the corresponding author upon reasonable request.
